# Chronic Disease Management Service Opportunities for Community Pharmacists During the COVID-19 Pandemic

**DOI:** 10.5888/pcd19.210280

**Published:** 2022-03-03

**Authors:** Gregory Dwyer, Adebola Popoola, Naomi Seiler, Nicole Therrien, Aaron Karacuschansky, Erika Fulmer, Katie Horton

**Affiliations:** 1 George Washington University School of Public Health, Washington, DC; 2 Centers for Disease Control and Prevention, Division for Heart Disease and Stroke Prevention, Atlanta, Georgia; 3 ASRT, Inc, Smyrna, Georgia

## Abstract

Health system disruption caused by the COVID-19 pandemic prompted public health professionals to reevaluate potential barriers and opportunities to community pharmacist provision of chronic disease management services and to identify opportunities for maximizing community pharmacists’ impact. Researchers conducted semistructured interviews with representatives from chronic disease prevention and pharmacy practice and policy organizations to identify key themes across multiple interviews and novel responses of interest. Interviewees described a lack of payment models to support pharmacist-provided chronic disease management services but noted opportunities for community pharmacists to demonstrate their value in offering services they are uniquely positioned to provide and to implement better workflow solutions. Successfully demonstrating pharmacists’ value and making the case for reimbursement from payors, as well as optimizing pharmacy workflow, are critical to maximizing pharmacists’ impact in chronic disease prevention and management.

**Summary**

**What is known on this topic?**

Community pharmacists, although uniquely situated to provide effective chronic disease management services, have encountered numerous barriers to providing these services both before and during the COVID-19 pandemic.

**What is added by this report?**

This research brief identifies barriers that prevent uptake of pharmacist-provided chronic disease management services and offers tactical and infrastructure opportunities to overcome them.

**What are the implications for public health practice?**

These insights may help public health practitioners leverage pharmacists’ unique skill sets and adapt to the evolving nature of community pharmacy practice in helping individuals with chronic disease achieve optimal health.

## Objective

The global disruption caused by the COVID-19 pandemic prompted policy makers, clinicians, and patients to consider how health care professionals deliver care, particularly as traditional avenues for care were overwhelmed with the COVID-19 response. Long before the pandemic, many pharmacy partners championed pharmacist involvement in providing chronic disease management (1), a task made even more critical by the pandemic (2). Given their proximity (3) to Americans, community pharmacists are well-positioned to fill gaps in chronic disease management services. Researchers sought to explore the role of community pharmacists in providing chronic disease management services to identify opportunities for maximizing community pharmacists’ impact.

## Methods

To understand the extent to which pharmacists are and could be used to provide services for chronic disease, academic researchers working with the Centers for Disease Control and Prevention’s Division for Heart Disease and Stroke Prevention interviewed pharmacists and chronic disease experts during nine 1-hour video interviews in June and July 2020. Interviewees represented 10 organizations in pharmacy policy and practice, including independent pharmacies, pharmacy chains, state boards of pharmacy, community pharmacists, and health-system pharmacists. Researchers also interviewed representatives of the American Heart Association. 

Using an interview guide, researchers conducted semistructured interviews that explored opportunities and barriers for community pharmacist provision of chronic disease management services before, during, and after the COVID-19 pandemic. Chronic disease management services, including medication reconciliation, chronic disease education and counseling, and medication management services (eg, medication therapy management and comprehensive medication management), were discussed.

Researchers reviewed transcripts of the interviews, identified key themes across multiple interviews as well as novel responses of interest, and developed a white paper describing 21 key findings from the interviews. All interviewees were given the opportunity to review the draft white paper to ensure that their comments were appropriately reflected. This article summarizes several novel findings from the white paper. Office on Management and Budget and institutional review board approvals were determined unnecessary and exempt, respectively.

## Results

All pharmacy-associated interviewees highlighted the lack of payment models to reimburse community pharmacists for chronic disease management as a barrier to community pharmacist provision of chronic disease management services. One pharmacist interviewee noted that, historically, pharmacists have provided chronic disease management services in an informal capacity without reimbursement. This arrangement was more feasible when pharmacy profits from other services, including dispensing, were more profitable, but as brick-and-mortar pharmacy profits have decreased (4), pharmacies have less capacity to provide services for which they do not receive reimbursement. Additionally, interviewees noted that pharmacists, particularly those working in rural areas, might be short-staffed or working alone and lack capacity to simultaneously fill prescriptions, administer vaccinations, and provide services related to chronic disease management such as medication therapy review and lifestyle and medication counseling.

Another interviewee stressed the need for community pharmacists to identify services that, given their education and skill set, they are exceptionally positioned to provide. Caring for patients who have multiple, complicated conditions and who are taking multiple medications with potential interactions is a role for which pharmacists are uniquely trained. The interviewee emphasized the importance of pharmacists focusing on the expertise they offer to chronic disease management to serve as valuable members of care teams.

Several interviewees noted that incorporating chronic disease management services and additional immunizations (including those for COVID-19) into the community pharmacy workflow may be challenging for some pharmacies due to lack of staffing and resources. Some pharmacies have made progress in improving practice workflow through medication synchronization, as well as the development of platforms to identify patients with chronic diseases and to guide pharmacists in providing chronic disease management services. Other innovations in practice infrastructure, such as use of centralized prescription processing facilities, have also been effective at allowing pharmacists to spend more time caring for patients who have chronic diseases.

## Discussion

The COVID-19 pandemic necessitated reimagining how health care systems deliver care to patients. Facilitating pharmacist provision of chronic disease management services involves ensuring that adequate staffing, technology, and workflow procedures are available to provide these services. COVID-19 has exacerbated existing workflow challenges, as many pharmacies deal with pandemic-related staffing shortages, COVID-19 testing, and vaccination.

Given the current environment, investing in health care delivery models that include and reimburse pharmacists for their unique skill set in providing chronic disease management services is critical for both COVID-19 and chronic disease management. In the longer term, evaluating the effects of payment models for pharmacist-provided chronic disease management services on both access to and quality of care will be important. In the near term, building the case for reimbursement may entail data-driven prioritization that focuses on those services most closely aligned with pharmacists’ unique skill sets.

Pharmacy practice is at a crossroads; pharmacies are choosing whether to pursue faster, cheaper business processes to compete with online retailers (4) or spend more resources to deliver quality care that patients cannot find through online retailers. Some pharmacies are choosing to invest in providing complex and enhanced services (5) while others are instead offering home delivery services (6) to rival the service of online retailers. The COVID-19 pandemic did not create these divergent paths, but it has accelerated the need to choose a direction.

The conclusions in this study are based on the perspectives of individuals working in pharmacy policy and practice. We did not consider the perspectives of other health care professionals who work with pharmacists to provide chronic disease management services. Future studies examining community pharmacists’ roles in chronic disease care from the perspective of these health care professionals may be valuable. Nevertheless, community pharmacists can play a critical role in delivering chronic disease management services.
